# LEAST as a novel prediction model of hepatocellular carcinoma development in patients with chronic hepatitis B: a multi-center study

**DOI:** 10.1186/s12916-025-04430-2

**Published:** 2025-11-03

**Authors:** Jingjing Song, Jie Li, Zhigang Ren, Wen Xie, Jinhua Shao, Xiaoxiao Zhang, Yang Zhou, Fajuan Rui, Xiaoqing Wu, Qiuling Wang, Zuxiong Huang, Chao Sun, Yuemin Nan

**Affiliations:** 1 Department of Traditional and Western Medical Hepatology, Hebei Medical University Third Hospital, Hebei Province, Shijiazhuang, 050051 China; 2 Hebei International Joint Research Center for Liver Cancer Molecular Diagnosis, Hebei International Science and Technology Cooperation Base, Hebei Province, Shijiazhuang, 050051 China; 3 Hebei Provincial Key Laboratory of Liver Fibrosis Mechanism Study in Chronic Liver Diseases, Hebei Province, Shijiazhuang, 050051 China; 4 Hebei Key Provincial Key Discipline of Gastroenterology for the 14th Five-Year Plan, Hebei Province, Shijiazhuang, 050051 China; 5 Department of Infectious Disease, Nanjing Drum Tower Hospital, Affiliated Hospital of Medical School, Nanjing University, Nanjing, 210008 Jiangsu China; 6 Department of Infectious Diseases, State Key Laboratory of Antiviral Drugs, Pingyuan Laboratory, The First Affiliated Hospital of Zhengzhou University, Zhengzhou, 450052 China; 7 Center of Liver Diseases, Beijing Ditan Hospital, Capital Medical University, Beijing, China; 8 Wuxi Hisky Medical Technologies Co., Ltd, Beijing, 100085 China; 9 Department of Hepatopancreatobiliary Surgery, Mengchao Hepatobiliary Hospital of Fujian Medical University, Fujian Province, Fuzhou, China; 10 Department of Gastroenterology and Hepatology, Tianjin Medical University General Hospital, Tianjin, China

**Keywords:** Hepatocellular carcinoma, Chronic hepatitis B, Prediction model, Nomogram, Liver stiffness measurement

## Abstract

**Background:**

Considering the heavy burden on healthcare resources owing to HBV infection and the broad feasibility of transient elastography techniques in China, we aimed to construct and corroborate a liver stiffness measure (LSM)-dictated prediction model concerning hepatocellular carcinoma (HCC) development among CHB patients.

**Methods:**

A retrospective cohort study was conducted, involving 713 consecutive patients with CHB. These patients were randomly assigned to the derivation (*n* = 534) and internal validation (*n* = 179) cohorts, respectively. Variable selection was optimized using the least absolute shrinkage and selection operator (LASSO) regression and subsequent multivariate Cox regression analysis. A corresponding nomogram was built and compared regarding discrimination, calibration, and risk stratification across the whole population. To further verify the generalizability of the predictive model, we integrated data from multiple external centers to construct two external validation cohorts for evaluation (*n* = 1084 and *n* = 623).

**Results:**

During a median follow-up duration of 57 months, 48 (8.99%) patients in the derivation cohort and 18 (10.06%) patients in the internal validation cohort developed HCC. Following the LASSO alongside Cox regression analyses, 5 variables were retained and constituted the LEAST model (LSM, age, albumin, sex, and platelet) and resulting nomogram. Our proposed model demonstrated sufficiently discriminative abilities to predict cumulative HCC development, as indicated by a time-dependent area under the curve (tdAUC) of 0.838 (95% CI 0.752–0.925), 0.898 (95% CI 0.851–0.944), and 0.907 (95% CI 0.856–0.959) over 3, 5, and 8 years, respectively. Nomogram-derived risk strata can appropriately identify patients at high risk of developing HCC. Our prediction model exhibited numerically the highest AUC compared to several previous scores. Moreover, the validity and generalizability of the LEAST model were verified in 2 independent external validation cohorts, confirmed in the calibration and stratification performance.

**Conclusions:**

The LEAST model could predict HCC development in CHB patients, facilitating the identification of high-risk patients who might benefit from enhanced surveillance or early therapy.

**Supplementary Information:**

The online version contains supplementary material available at 10.1186/s12916-025-04430-2.

## Background

Primary liver cancer remains a prevalent and significant public health concern worldwide. According to the most recent global cancer statistics 2022, it is estimated that the incidence and mortality of liver malignancy rank 6th and 3rd, respectively, with 865,273 new cases and 757,906 deaths globally [1]. In mainland China, these figures rank fifth and second, respectively, as indicated by 367,657 new cases and 316,544 deaths. Hepatocellular carcinoma (HCC) accounts for the predominant type of primary liver cancer (i.e., 90% of all cases). HBV infection serves as the commonest risk factor associated with the onset and progression of HCC, while 50%–60% of all incidental HCC cases can be pathogenically attributable to chronic hepatitis B (CHB) [2, 3]. From a practical perspective, effective HCC management is challenging because the majority of patients are diagnosed at advanced stages, giving rise to poor prognosis and unfavorable outcomes. Currently, the 5-year survival rate of HCC has been reported to be merely 14.1% in our country owing to the insidiousness but rapid deterioration of this aggressive entity [4]. Accordingly, accurate, swift, and prompt identification, diagnosis, and therapy are cornerstones to improve HCC survival status and related outcomes like health-related quality of life [5, 6]. Therefore, it is crucial to develop and verify risk stratification systems pertinent to disease progression in subjects with HBV infection to improve prognosis.

Looking into the existing literature, many models have been constructed in the context of diversely targeted populations, different geographical areas, and distinct therapeutic regimens, incorporating heterogeneous risk factors and showing moderately to well predictive utility alongside prognostic performance [7–12]. However, one drawback should be acknowledged, since those models have regarded the presence of liver cirrhosis as a significant predictor of HCC, whose definite diagnosis relies on mixed workups like radiology, endoscopy, and biochemical findings (e.g., thrombocytopenia, hypoalbuminemia, jaundice) rather than histological confirmation. This may lead to underestimated performance, and in consequence, delayed HCC detection and treatment.

Transient elastography (TE) defined-liver stiffness measurement (LSM) is a reliable, reproducible, and noninvasive toolkit to identify subjects experiencing liver fibrosis or cirrhosis [13]. Aligning with a marked increase in the cumulative HCC incidences as indicated by a higher LSM score, this modality has been widely adopted to assess and stratify HCC risk with remarkable performance [9, 11, 14]. Given the heavy burden on healthcare resources owing to HBV infection and the broad feasibility of TE techniques in China, we herein hypothesize that it is tempting to generate a novel and readily available LSM-dictated model, in hopes of enhancing surveillance and prevention in patients with CHB who are prone to dramatic HCC aggravation. Furthermore, a sizable derivation cohort and two independent external validation cohorts have been used for ascertainment in this study.

## Methods

### Patients

This was a retrospective study by consecutively enrolling patients between January 2013 and December 2023, who were followed up until December 2024. The inclusion criteria consisted of (1) persistent serum HBsAg presence for > 6 months, (2) completed follow-up data for ≥ 6 months, and (3) at least two follow-up visits. The exclusion criteria comprised (1) coinfection with hepatitis A/C/D/E virus or human immunodeficiency virus; (2) prior history of HCC or other malignancies at enrollment; (3) confirmed HCC, deaths, or receiving liver transplant < 6 months of the enrollment; (4) lost to regular follow-up; (5) incomplete clinical and/or laboratory data; (6) without verified LSM; or (7) pregnancy or lactation. Notably, the external validation datasets conformed to the same inclusion/exclusion criteria (external validation cohort 1 [EV-1]: individuals from Mengchao Hepatobiliary Hospital of Fujian Medical University; external validation cohort 2 [EV-2]: individuals from Beijing Ditan Hospital of Capital Medical University, Nanjing Drum Tower Hospital, and the First Affiliated Hospital of Zhengzhou University). This study aligned with the ethical standards of the Declaration of Helsinki and was approved by the Ethics Committee of Hebei Medical University Third Hospital (W2024-004–1). This study was conducted in accordance with the Transparent Reporting of a Multivariable Prediction Model for Individual Prognosis or Diagnosis (TRIPOD) guidelines for the development and validation of prediction models [15].

### Clinical/laboratory evaluation and follow-up

Clinical data, including age, sex, alcohol use, family history of liver cirrhosis/HCC, comorbidities, and a range of laboratory parameters, were retrospectively collected at baseline. All patients underwent periodic surveillance of laboratory tests, including routine blood chemistry, serum HBV DNA level, and other serologic viral markers, at a 3- to 6-month interval. Meanwhile, serum alpha-fetoprotein (AFP) level and abdominal ultrasound were recorded every 6 months to screen for possible hepatic decompensation or HCC. Liver cirrhosis was diagnosed to fulfill the respective liver histology, clinical features, and radiological evidence or their combinations. The primary outcome indicated development of HCC in terms of histological or radiological findings [16].

LSM was performed using a liver TE scanner (iLivTouch, Wuxi HISKY Medical Technologies Co., Ltd., China). Although a high correlation of LSM between operators, interobserver variability in TE should not be negligible [17, 18]. In this study, all measurements were performed by experienced operators trained for TE with over 100 operations. The operators underwent professional operation training and conducted practical operation demonstrations under guidance, and were assessed to obtain the training certificate issued by the manufacturer. According to the manufacturer, the result was considered reliable only if at least 10 successful acquisitions were obtained, and the interquartile range-to-median ratio was less than 0.3 [19]. Throughout the study, ongoing monitoring of LSM data was conducted. All measurements were reviewed by investigators to ensure continued adherence to protocol. The patients were scanned in the supine or lateral position while hugging their head with the right hand to maximize the intercostal space. Under ultrasound guidance, the 7th, 8th, or 9th intercostal space from the right anterior axillary line to the midaxillary line was selected as the detection point.

### Statistical analysis

Statistical analysis was employed using the R software (version 4.3.2; https://www.R-project.org). Using a theoretical ratio of 3:1, patients were randomly split into the derivation cohort, responsible for screening variables and constructing the model, and the internal validation cohort, which was used to verify the results obtained. At the same time, data from two independent external validation cohorts were applied to testify the generalizability and validity of the proposed model. Categorical variables are presented as the number and percentage. On the other hand, continuous variables are presented as the mean (standard deviation [SD]) or the median (interquartile range [IQR]) as appropriate according to the normal distribution attributes. The predictive performance of the model was assessed using the receiver operating characteristic (ROC) curve.

We performed a least absolute shrinkage and selection operator (LASSO) regression algorithm to select features initially in the derivation cohort [20]. The tenfold cross-validation method was employed to choose the optimal regularization parameter λ (minimizes the binomial deviance: λ-min = 0.01). To balance the model’s complexity and performance, 7 variables with nonzero coefficients were finally retained according to the critical value setting as λ−1se = 0.03. The proportional hazards assumption was checked using the Schoenfeld residuals, and all selected variables exhibited equally without time-course fluctuation. By incorporating these LASSO-selected variables, a multivariate Cox proportional hazards regression was utilized to identify independent risk factors of HCC, which were finally visualized in the resulting nomogram.

We also assembled several validation approaches to evaluate the prediction model’s accuracy across the derivation and validation cohorts. Regarding the predictive performance, we evaluated and compared both their discrimination and calibration abilities. The discriminative ability of our proposed nomogram was estimated in terms of Harrell’s concordance index (c-index), along with the time-dependent area under the curve (tdAUC) at 3, 5, and 8 years. On the other hand, the model’s consistency was assessed using the calibration curve, as shown in plots of estimated versus observed probabilities and the Hosmer–Lemeshow goodness of fit test [21]. Additionally, the X-tile software was employed to identify three risk strata associated with HCC development based on the total score derived from the nomogram, categorizing the participants into the low-, intermediate-, and high-risk groups. The Kaplan–Meier method for each risk group at different stages was constructed, and the cumulative HCC development between groups was analyzed using the log-rank test. A *P* value of < 0.05 was regarded as statistically significant.

## Results

### Baseline characteristics of the study cohort

**Fig. 1:**
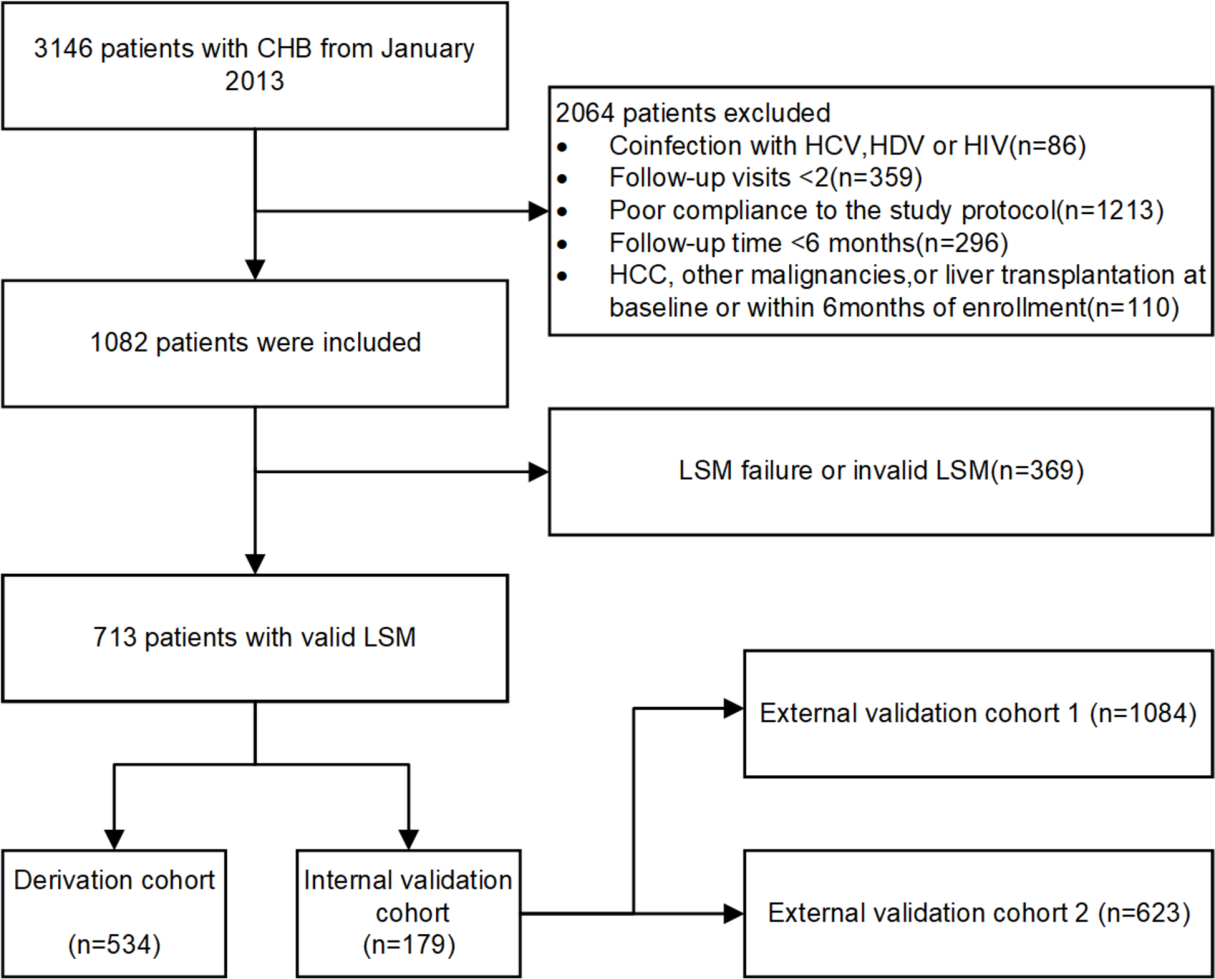
Flowchart of the current study. CHB, chronic hepatitis B; HCC, hepatocellular carcinoma; HCV, hepatitis C virus; HDV, hepatitis D virus; HIV, human immunodeficiency virus; LSM, liver stiffness measurement

Of the 3146 patients with positive HBsAg during the study period, some were excluded according to the criteria above (*n* = 2064) and because of failed or invalid LSM (*n* = 369). Finally, a total of 713 patients with complete LSM alongside clinical information were recruited in the analysis. After random assignment, 534 and 179 patients were included in the derivation and validation cohorts, respectively (Fig. 1); the clinical features between these two cohorts were comparable (all *P* > 0.05) (Table 1). In the derivation cohort and internal validation cohort, 48 patients (8.99%) and 18 patients (10.06%) developed HCC during a median follow-up of 57 months (IQR, 30–85). In the EV-1 and EV-2 cohorts, 119 patients (10.98%) and 46 patients (7.38%) developed HCC, respectively (Additional file 1: Table S1).

**Table 1:** Baseline characteristics of patients with CHB

	Total (*n* = 713)	Derivation cohort (*n* = 534)	Validation cohort (*n* = 179)	*P*
HCC (%)	66 (9.26)	48 (8.99)	18 (10.06)	0.782
Male (%)	419 (58.77)	313 (58.61)	106 (59.22)	0.957
Age (years)	46.00 (36.00, 55.00)	46.00 (36.00, 55.00)	46.00 (36.50, 55.00)	0.870
Fatty liver (%)	79 (11.08)	56 (10.49)	23 (12.85)	0.463
Hypertension (%)	95 (13.32)	76 (14.23)	19 (10.61)	0.269
Hyperlipidemia (%)	151 (21.18)	113 (21.16)	38 (21.23)	1.000
Diabetes (%)	126 (17.67)	92 (17.23)	34 (18.99)	0.672
CVD (%)	29 (4.07)	23 (4.31)	6 (3.35)	0.733
Alcohol use (%)	133 (18.65)	99 (18.54)	34 (18.99)	0.981
Family history (%)	292 (40.95)	215 (40.26)	77 (43.02)	0.575
Antiviral (%)	331 (46.42)	238 (44.57)	93 (51.96)	0.103
WBC (10^9^/L)	4.83 (3.59, 5.97)	4.88 (3.64, 6.09)	4.51 (3.45, 5.82)	0.081
HGB (g/L)	139.00 (121.60, 154.00)	139.00 (121.65, 153.00)	138.60 (121.50, 156.90)	0.407
PLT (10^9^/L)	150.30 (92.00, 212.00)	152.00 (93.78, 212.00)	146.00 (86.00, 209.45)	0.584
ALB (g/L)	43.30 (36.25, 47.20)	43.62 (36.29, 47.30)	42.90 (35.91, 46.88)	0.460
ALT (U/L)	39.00 (23.00, 83.00)	39.00 (23.00, 75.75)	41.00 (23.00, 88.00)	0.340
AST (U/L)	36.00 (24.00, 66.00)	36.00 (24.00, 64.75)	36.00 (24.00, 68.00)	0.538
TB (µmol/L)	18.20 (12.70, 30.30)	18.04 (12.72, 30.30)	18.60 (12.65, 30.38)	0.969
ALP (U/L)	75.00 (57.00, 95.00)	74.00 (56.00, 96.00)	77.00 (63.00, 92.50)	0.176
GGT (U/L)	35.00 (22.00, 65.00)	34.00 (21.00, 65.00)	37.00 (24.00, 66.50)	0.447
TBA (µmol/L)	8.20 (3.10, 30.30)	8.25 (2.92, 28.37)	7.80 (3.75, 36.30)	0.294
LDH (U/L)	177.00 (152.00, 214.29)	177.00 (151.58, 214.36)	177.00 (152.49, 213.01)	0.957
TC (mmol/L)	3.88 (3.24, 4.59)	3.88 (3.25, 4.59)	3.88 (3.21, 4.58)	0.690
TG (mmol/L)	0.96 (0.70, 1.30)	0.96 (0.70, 1.27)	0.96 (0.70, 1.34)	0.274
HDL (mmol/L)	1.18 (0.96, 1.42)	1.18 (0.96, 1.43)	1.18 (0.95, 1.41)	0.802
LDL (mmol/L)	2.33 (1.80, 2.87)	2.33 (1.78, 2.85)	2.33 (1.85, 2.96)	0.507
GLU (mmol/L)	5.31 (4.88, 5.89)	5.31 (4.89, 5.86)	5.34 (4.86, 6.06)	0.350
CREA (µmol/L)	64.89 (55.10, 73.97)	64.89 (55.01, 73.96)	64.89 (55.30, 74.08)	0.856
UA (µmol/L)	299.00 (226.00, 364.00)	301.00 (222.75, 366.75)	287.00 (232.50, 354.00)	0.546
PT (s)	12.10 (11.20, 13.70)	12.10 (11.25, 13.80)	12.10 (11.20, 13.45)	0.466
APRI	0.84 (0.42, 1.60)	0.80 (0.42, 1.61)	0.98 (0.42, 1.59)	0.362
FIB-4	1.92 (1.00, 4.07)	1.93 (0.98, 4.04)	1.87 (1.09, 4.14)	0.717
HBeAg (%)	361 (50.63)	263 (49.25)	98 (54.75)	0.235
HBV DNA (log_10_ IU/mL)	3.23 (2.32, 5.97)	3.08 (2.18, 5.87)	3.55 (2.40, 6.16)	0.354
AFP (ng/mL)	3.47 (2.26, 6.57)	3.50 (2.30, 6.59)	3.35 (2.16, 6.51)	0.875
BMI (kg/m^2^)	25.43 (22.84, 27.41)	25.46 (22.65, 27.43)	25.39 (23.11, 26.88)	0.695
LSM (kPa)	10.54 (6.90, 15.74)	10.50 (6.80, 15.55)	10.86 (7.05, 16.28)	0.274
UAP (dB/m)	242.34 (217.75, 270.00)	242.00 (216.90, 268.41)	242.42 (219.57, 276.00)	0.193

*CHB*, chronic hepatitis B; *HCC*, hepatocellular carcinoma; *CVD*, cardiovascular disease; *WBC*, white blood cell; *HGB*, hemoglobin; *PLT*, platelet; *ALB*, albumin; *ALT*, alanine transaminase; *AST*, aspartate aminotransferase; *TB*, total bilirubin; *ALP*, alkaline phosphatase; *GGT*, gamma-glutamyl transferase; *TBA*, total bile acid; *LDH*, lactate dehydrogenase; *TC*, total cholesterol; *TG*, triglyceride; *HDL*, high-density lipoprotein cholesterol; *LDL*, low-density lipoprotein cholesterol; *GLU*, glucose; *CREA*, creatinine; *UA*, uric acid; *PT*, prothrombin time; *APRI*, aspartate aminotransferase to platelet ratio index; *FIB-4*, Fibrosis 4 score; *AFP*, alpha-fetoprotein; *BMI*, body mass index; *LSM*, liver stiffness measurement; *CAP*, controlled attenuation parameter

### Variable selection

**Fig. 2:**
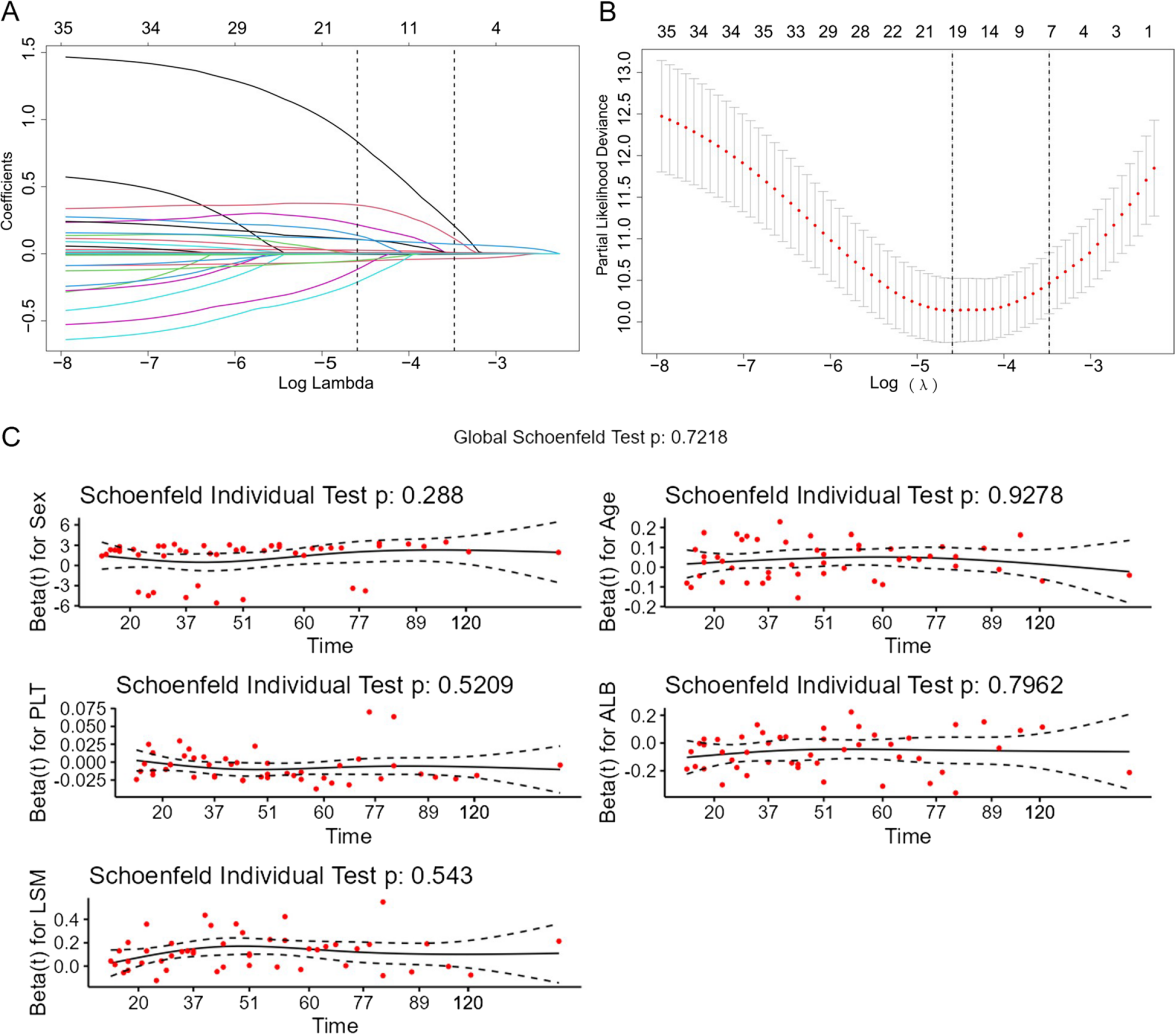
Variable selection in terms of the LASSO regression. **A** LASSO coefficient profiles (*y*-axis) of 35 clinical features. The top *x*-axis shows the number of variables. The lower *x*-axis shows the log (λ). **B** The optimal penalization coefficient (λ) in the LASSO model was identified with tenfold cross-validation, which shows the binomial deviance of the LASSO regression model for different λ values, used to select the optimal one. **C** Proportional variance analysis results for each variable in the multivariate Cox regression model. The *x*-axis represents time, while the *y*-axis represents Schoenfeld residuals. Each variable corresponds to a curve, with the solid line indicating the trend of residuals over time and the dashed line representing the 95% confidence interval. LASSO, least absolute shrinkage and selection operator

In response to LASSO regression selection, 7 variables remained significantly predictive of HCC, including sex, age, alcohol use, platelet (PLT), albumin (ALB), AFP, and LSM value (Fig. 2). Incorporation of these 7 variables in a multivariate Cox proportional hazards model gave rise to 5 variables which were statistically independent risk factors of HCC development and used to construct the nomogram (Additional file 2: Fig. S1 and Additional file 1: Table S2), The variables were LSM value (HR 1.129, 95% CI 1.083–1.177, *P* < 0.001), PLT (HR 0.994, 95% CI 0.988–0.999, *P* = 0.021), sex (HR 3.235, 95% CI 1.541–6.790, *P* = 0.002), and age (HR 1.037, 95% CI 1.010–1.064, *P* = 0.007) in addition to ALB (HR 0.940, 95% CI 0.899–0.983, *P* = 0.006) (Table 2).

**Table 2:** Multivariate Cox regression analyses of risk factors in the derivation cohort

Variable	HR	95% CI	*P*
Sex	3.235	1.541–6.790	0.002
Age	1.037	1.010–1.064	0.007
PLT	0.994	0.988–0.999	0.021
ALB	0.940	0.899–0.983	0.006
LSM	1.129	1.083–1.177	< 0.001

*ALB*, albumin; *PLT*, platelet; *HR*, hazard ratio; *CI*, confidence interval; *LSM*, liver stiffness measurement

### Establishment and evaluation of our proposed nomogram to predict HCC development

**Fig. 3:**
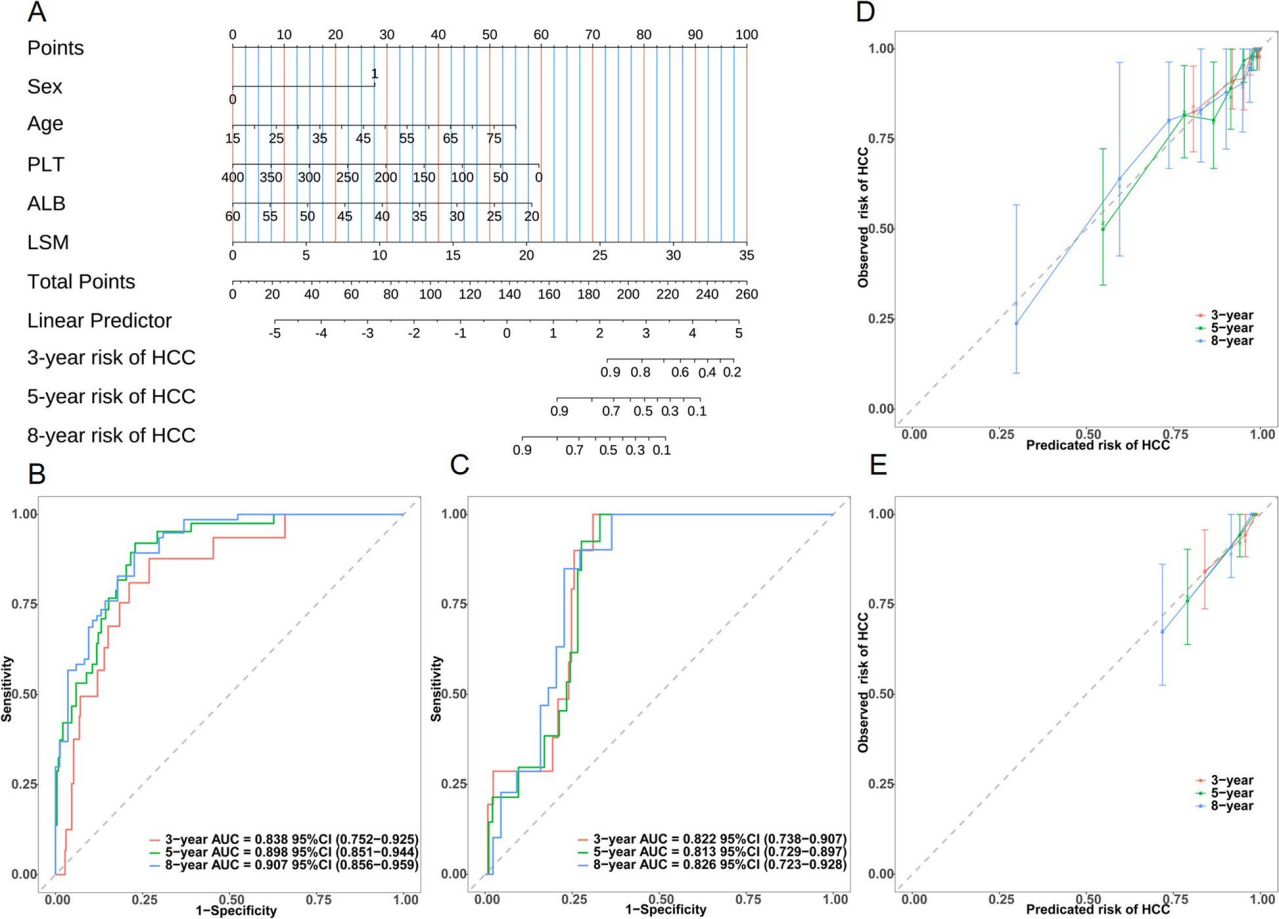
Establishment of the LEAST model-dictated nomogram for patients with CHB and evaluation of its performance in the derivation and internal validation cohort. **A** A nomogram was plotted to predict HCC development among patients with CHB. For instance, a patient with CHB visited the hospital. In the nomogram model, each variable was assigned a specific score on the rating scale. So, sex (male) = 27.6 points, age (60 years old) = 38.1 points, PLT (200*10^9^/L) = 29.6 points, ALB (30 g/L) = 43.7 points, and LSM (20 kPa) = 57.2 points, total score = 27.6 + 38.1 + 29.6 + 43.7 + 57.2 = 196.2 points. The predicted probability of HCC was obtained by summing the scores for each variable and drawing a vertical line down the total score. This patient is categorized as an intermediate-risk individual concerning the occurrence of HCC. Furthermore, using the nomogram model, we can obtain the predicted probabilities of the patient developing HCC within 3 years, 5 years, and 8 years, respectively, as 86.9%, 66.1%, and 41.7%. **B**, **C** Time-dependent area under the curve of the LEAST model at 3-, 5-, and 8-year follow-up in the derivation cohort and the internal validation cohort. **D**, **E** Calibration of the proposed nomogram at 3-, 5-, and 8-year follow-up in the derivation cohort and the internal validation cohort. ALB, albumin; CHB, chronic hepatitis B; HCC, hepatocellular carcinoma; LSM, liver stiffness measurement

The final prediction model, termed the LEAST model, included five predictors: LSM value, age, ALB, sex, and PLT. Accordingly, a corresponding nomogram was established: each variable was assigned a specific point value, and the total score was calculated by summing the points from all contributing features to estimate the probability of HCC development (Fig. 3A).

### Comparison and calibration of our proposed nomogram

Our results indicated that the LEAST model exhibited sufficient prediction accuracy at 3, 5, and 8 years of follow-up. The c-index was 0.859 (95% CI 0.810–0.908) and 0.828 (95% CI 0.759–0.897) in the derivation cohort and internal validation cohort, respectively. The AUCs for the 3-year, 5-year, and 8-year in the derivation cohort were 0.838 (95% CI 0.752–0.925), 0.898 (95% CI 0.851–0.944), and 0.907 (95% CI 0.856–0.959), respectively (Fig. 3B). In the internal validation cohort, the AUCs for 3-year, 5-year, and 8-year concerning HCC development were 0.822 (95% CI 0.738–0.907), 0.813 (95% CI 0.729–0.897), and 0.826 (95% CI 0.723–0.928), respectively (Fig. 3C). The calibration curves showed a close correspondence between the predicted and observed survival probabilities in both cohorts, suggesting constant predictive ability of the LEAST model (Fig. 3D, E). The Hosmer–Lemeshow goodness of fit test showed that our proposed nomogram was well-calculated (χ^2^ = 4.074, *P* = 0.850).

### Risk stratification according to our proposed nomogram

**Fig. 4:**
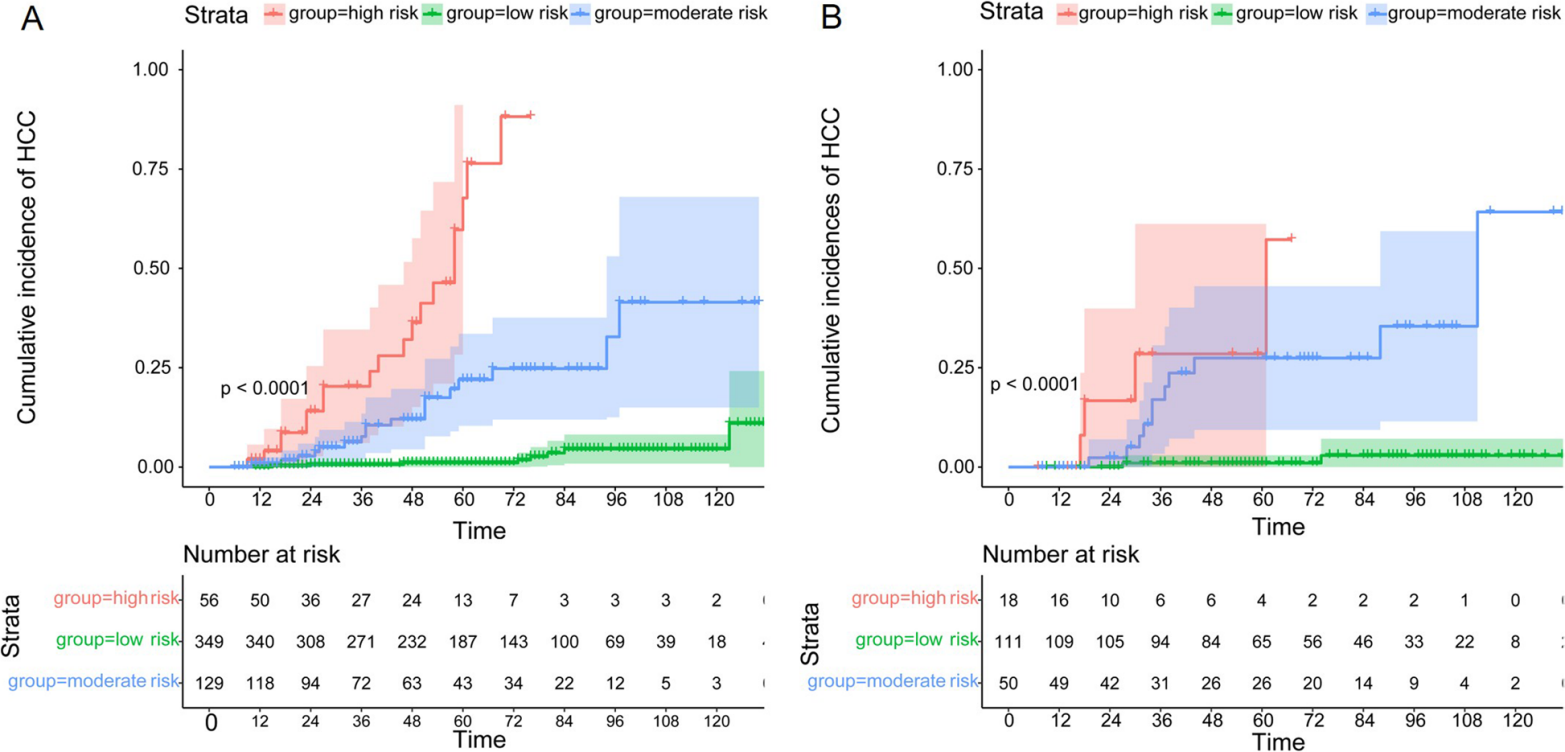
Risk stratification of HCC development based on the LEAST model-dictated nomogram in the derivation cohort (**A**) and the internal validation cohort (**B**) in terms of the Kaplan–Meier method

Applying joint nomogram-defined total scores and X-tile processing, the derivation cohort was further categorized into tertiles with low risk (< 160.7 points), intermediate risk (160.7–191.2 points), and high risk (> 193.2 points) of HCC development (Additional file 2: Fig. S2). Kaplan–Meier curves implied that our proposed nomogram exhibited a good risk stratification concerning the cumulative HCC development (log-rank test *P* < 0.0001, Fig. 4A). Notably, a similar trend was evident in the internal validation cohort (Fig. 4B). Moreover, rounded cut-offs (low risk < 160 points, high risk > 193 points) consistently stratified both external validation cohorts into distinct HCC development risk groups (log-rank test *P* < 0.0001), partially supporting practical convenience (Additional file 2: Fig. S3 and Additional file 2: Fig. S4).

### Comparison with precursors and external validation

**Fig. 5:**
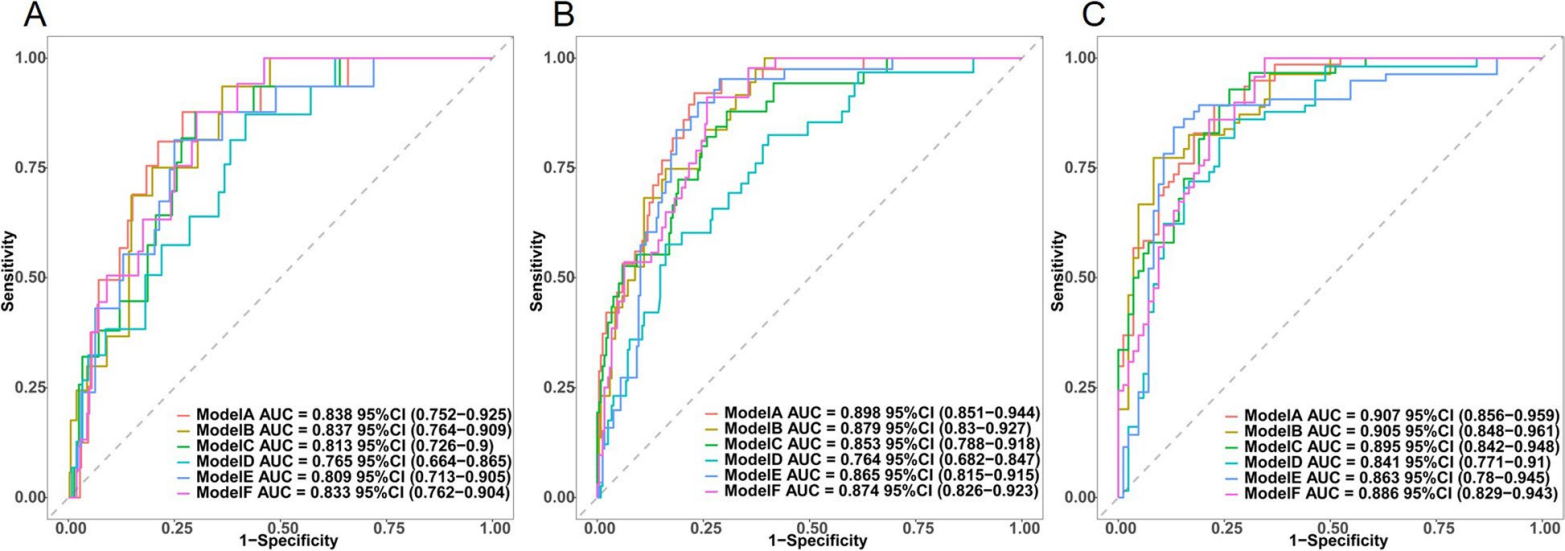
Comparison of ROC curves at 3-, 5-, and 8-year follow-up to predict HCC development between multiple models. Model A: LEAST, model B: REAL-B, model C: mREACH-B, model D: AGED, model E: mPAGE-B, model F: LSM-HCC. HCC, hepatocellular carcinoma; ROC, receiver operating characteristic

To ascertain the validity of the LEAST model, discriminative abilities as indicated by AUCs were compared with the 5 existing HBV-related HCC risk scores [Real-world Effectiveness for HBV (REAL-B), modified REACH-B (mREACH-B), AGED, Liver Stiffness Measurement HCC (LSM-HCC), and modified PAGE-B (mPAGE-B) scores]. Compared with those previously established models, the LEAST model demonstrated well-discriminative performance. As depicted in Fig. 5, this novel model had numerically the highest AUCs across the 3-, 5-, and 8-year follow-up relative to their precursors. We have illustrated the differences in discriminative abilities of all prediction models as a supplementary file for clarification (Additional file 1: Table S3).

**Fig. 6:**
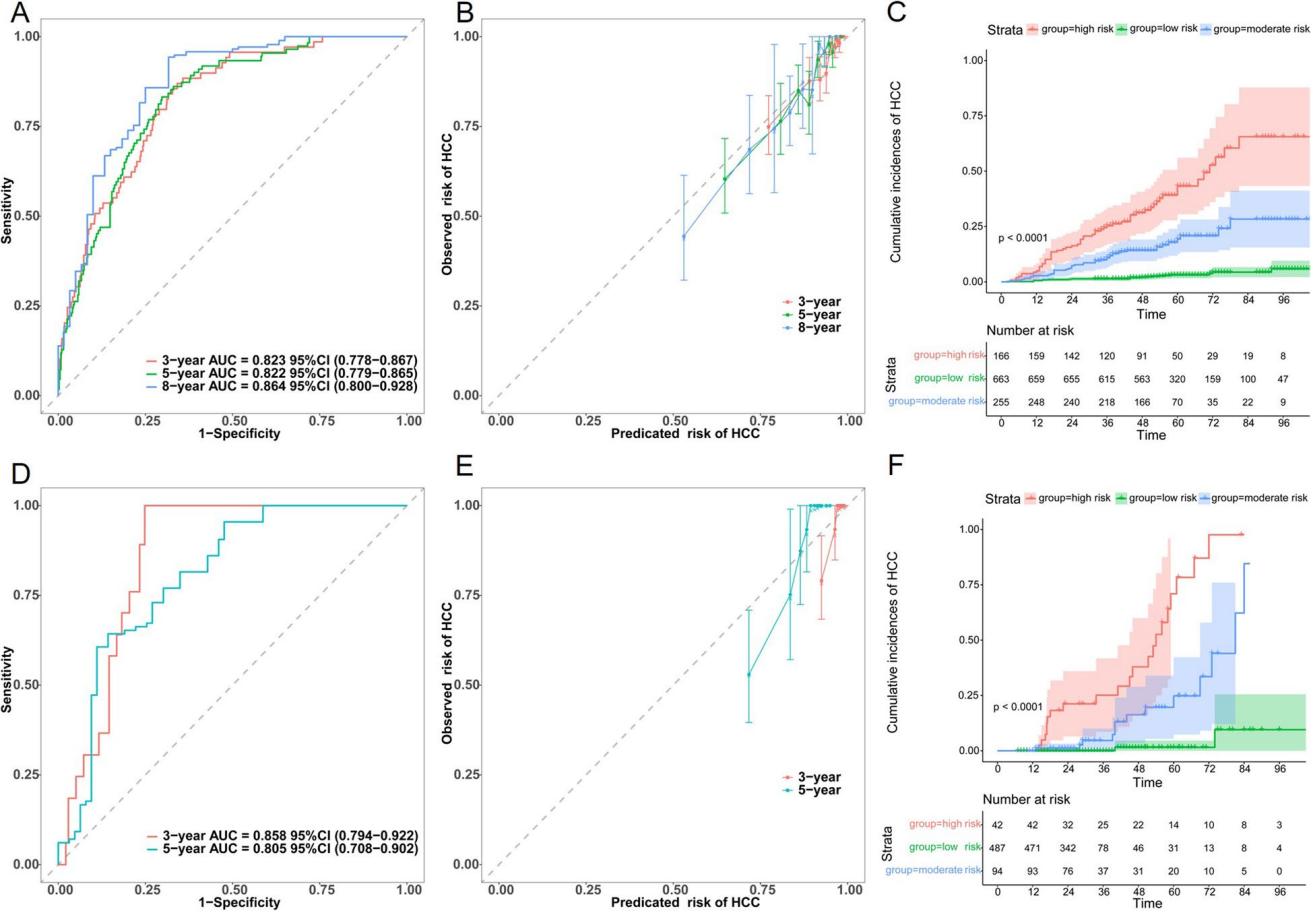
Assessment of discriminative ability, calibration, and risk stratification in the EV-1 (**A**–**C**) and EV-2 (**D**–**F**). EV-1, external validation cohort 1; EV-2, external validation cohort 2

Regarding generalizability, 2 external validation cohorts were tested, with cumulative HCC development in 7.38% and 10.98% patients, respectively. Both EV-1 and EV-2 showed moderately discriminative abilities on the AUCs, conforming to respective c-indexes of 0.807 (95% CI 0.772–0.842) and 0.905 (95% CI 0.862–0.948) (Fig. 6A/D). Other performances, like calibration (Fig. 6B/E) and risk stratification (Fig. 6C/F), were also corroborated.

## Discussion

In this study, we constructed and widely corroborated a novel prediction model, designated as the LEAST, which was closely related to HCC development from a sizable cohort. Moreover, the resulting nomogram exhibited sufficient capabilities concerning discrimination, calibration, and risk stratification across the entire derived and verified populations. The superior performance of our LSM-based LEAST model relative to numerous existing scores highlights the clinical utility of incorporating this surrogate quantitative biomarker of fibrotic burden for predicting the risk of HCC development.

There has been a surge to establish prediction models/risk scores pertinent to the development of HCC and compare their performance in the setting of CHB infection. However, the results should be interpreted cautiously, and conclusions are elusive owing to marked heterogeneity regarding the targeted populations’ ethnicity, baseline characteristics, and antiviral prescription [22]. Taking into account a considerable proportion of individuals with HBV infection remained unidentified or poor adherence to persistent antiviral therapy, therefore, the majority of subjects are diagnosed with HCC at an advanced stage, missing the optimal opportunity for surgical treatment. Of note, another concerning factor is the rapidly aging population, which continues to increase the HCC burden [1]. In this respect, recent studies have unraveled that the prognosis of HCC remains unfavorable, with a 5-year OS rate ranging from 10 to 19%, as indicated by the CONCORD program [23]. By contrast, the 5-year OS rate was reported to approach as high as 86.2% in early-stage HCC, highlighting the importance of early detection, prompt diagnosis, and accurate prognostication.

Regarding our proposed model, several variable selection modalities have identified the LSM, age, ALB, sex, and PLT as independent predictors of HCC development. Older patients exhibited 3.6 to 8.3 times higher risk of cumulative HCC presence [24]. On the other hand, sex (male predominance) and decreased serum ALB and PLT have also been incorporated in several LSM-based models, closely connected to genetic predisposition, life behavior, and the magnitude of portal hypertension on account of underpinning liver cirrhosis [11, 25, 26]. Notably, a steady performance of the LEAST model in long-term prognosis may raise concerns from the practitioner, in particular, its AUC has attained the numerically highest at 8 years of follow-up. Several possible reasons may account for these additional benefits. First, in the era of potent antiviral regimens such as entecavir and tenofovir with a genetic barrier to resistance, long-term treatment may modify the natural history of CHB infection and curtail the prognostic significance of viral-load factors. Nowadays, it is easy to achieve a complete virological response even in patients with high pretreatment viral load, but the risk of developing HCC cannot be obviated or underestimated. This also partially explains the poor performance of the AGED model across the study timeframe, which incorporated both HBV DNA and HBeAg, but from a data set established around 30 years ago (i.e., 1996) [27]. Second, LSM is a promising and cost-effective surrogate that more accurately and quantitatively captures the magnitude of fibrotic alterations. Moreover, the LSM value was found to increase in parallel with the liver fibrosis status, implicated in the HCC progression [28]. It is highlighted that even subjects with low fibrotic burden cannot avoid the risk of developing HCC [14, 25]. Third, the diagnosis of liver cirrhosis relies on a combination of clinical symptoms/signs, laboratory results, and radiological examinations, along with the “gold standard” liver biopsy. However, the former workups may omit patients with insidious symptoms, while the latter modality tends to be less accepted by subjects due to its invasive nature and possible life-threatening complications [29]. Fourth, the LEAST model may address a delicate balance between complexity and validity. Specifically, the five components in our prediction model cover those in the mPAGE-B score, which is simpler than the REAL-B score by dropping AFP. Actually, a baseline AFP > 10 ng/mL was ascertained to be independently predictive of HCC in the REAL-B score. Furthermore, this metric reflecting necroinflammation and hepatocarcinogenesis also fell out in the mREACH-B score, probably due to low baseline AFP levels in both investigated cohorts (mREACH-B model: median 3.00 ng/mL; LEAST model: 3.47 ng/mL). When looking at the alcohol use, whose falling out may be ascribed to a significant sex disparity concerning this habit. In the derivation cohort, males account for 99% of the population in alcohol use, directly impacting its independence as a risk factor. For instance, a large-scale screening program implicated that habitual alcohol consumption was associated with HCC only in males [30]. Moreover, another explanation is that the recruited population may underrepresent specific subgroups owing to our stringent exclusion criteria. Specifically, 1213 and 369 subjects were ruled out due to poor compliance and invalid TE procedure, respectively. This selection bias shapes the investigated cohorts to a “healthier” and highly compliant phenotype. Actually, alcohol consumption may serve as an indirect indicator of unhealthy dietary habits and is under-reported given social stigma, weakening the relationship between certain behaviors and the risk of HCC [31]. Keeping this in mind, several methodological refinements can be employed, including but not limited to a time-based split of recruited subjects to mimic a real-world temporal scenario and evaluate prospective performance, and the adoption of sensitive biomarkers of habitual alcohol intake based on metabolomics rather than self-reported assessments [32]. Fifth, the absence of diabetes as a significant risk factor in the LEAST model is consistent with the study mentioned earlier, but warrants further verification. A recent study stated that even an increased diabetes prevalence of 24.3% may not be enough to influence the performance of risk scores concerning HCC development in the entire CHB population [33]. Considering the increasing trend of diabetes prevalence, practitioners are expected to encounter more patients with concurrent CHB and diabetes in the future.

Our study has some strengths. First, the sizable study populations and relatively long follow-up period (median > 5 years, maximum of approximately 10 years) may have increased this study’s statistical power and feasibility. Second, inclusion of multiple variables in the multivariate analysis was deemed to effectively adjust for potential confounders and enhance the accuracy of our prediction model, thereby providing more reliable evidence to make clinical decisions. Third, precursors have addressed several variables, such as family history, personal history, sex, age, ALB, and AFP; these factors and HBV DNA, which reflects viral load, were fully considered. The comprehensive evaluation of these factors and the interaction of mixed laboratory indicators enhanced the discrimination of our model. Fourth, most of the targeted population in prior reports regarding HCC-prediction scores originated from the USA, South Korea, or the local region of China (e.g., Hong Kong). Still, our research has focused on the mainland Chinese population experiencing CHB with broad geographical distribution, as indicated by the representativeness in the two independent external validation cohorts.

Several limitations should be acknowledged. First, our proposed model applies only baseline information rather than dynamic on-treatment profiles and lacks decision-curve analysis-determined clinical utility, desirable for future improvement and refinement. Second, since the data were generated from a mixed cohort (i.e., treatment naïve and treated at initial enrollment), it is uncertain whether the findings may apply to subjects with universal antiviral treatment. It is noted that the predictability of extant HCC risk models declined with the antiviral treatment prolongation, thus an amendment to the original model and risk strata may be required [34]. Third, potential inter-institutional variability may result in a confounding impact on our results, although the TE scanner has already been performed by well-trained and qualified staff. Fourth, the cut-offs derived from X-tile for risk stratification may over-fit the HCC population, thereby reducing their clinical significance. Lastly, the follow-up period in the EV-2 cohort was limited to 5 years, thus partially curtailing the statistical power.

## Conclusions

In conclusion, the LSM value was positively associated with the risk of developing HCC. The LEAST model and resulting nomogram had sufficiently discriminative abilities for HBV-related HCC development and can appropriately identify high-risk settings. Future studies are warranted to corroborate our proposed model and combine other metrics, like imaging techniques, liquid biopsy analyses, and omics profiles, to formulate individualized treatment and surveillance strategies.

## Supplementary Information

Additional file 1: Tables S1–S3. Table S1 Baseline characteristics of the external validation cohort. Table S2 Goodness of fit and collinearity diagnosis of model variables. Table S3 The differences in discriminative ability between all the predictive models and the LEAST model.

Additional file 2: Figures S1–S4. Fig. S1 (A) Variables selected by the LASSO regression. (B) Forest plots for multivariate analyses of risk factors concerning HCC development. LASSO, least absolute shrinkage and selection operator; HCC, hepatocellular carcinoma. Fig. S2 Applying the thresholds established by X-tile on the nomogram, the derivation cohort was divided into three risk categories. ALB, albumin; PLT, platelet; HR, hazard ratio; CI, confidence interval; LSM, liver stiffness measurement. Fig. S3 Kaplan–Meier curves for the external validation cohort 1, after dichotomizing the cohort using the risk-stratification cut-off value rounded to the nearest integer (low risk < 160 points, intermediate risk 160–193 points, high risk > 193 points). HCC, hepatocellular carcinoma. Fig. S4 Kaplan–Meier curves for the external validation cohort 2, after dichotomizing the cohort using the risk-stratification cut-off value rounded to the nearest integer (low risk < 160 points, intermediate risk 160–193 points, high risk > 193 points). HCC, hepatocellular carcinoma. Graphical Abstract—This graphical abstract illustrates the overall workflow for developing and validating a hepatocellular carcinoma prediction model. The process begins with a baseline study of a chronic hepatitis B cohort, followed by the construction of a statistical model, and concludes with successful validation across multiple independent external cohorts. 

## Data Availability

The datasets used and/or analysed during the current study are available from the corresponding author on reasonable request.

## Abbreviations

AFP: Alpha-fetoprotein
APRI: Aspartate aminotransferase to platelet ratio index
AVT: Antiviral therapy
CHB: Chronic hepatitis B
CI: Confidence interval
CVD: Cardiovascular disease
FIB-4: Fibrosis 4 score
HBV: Hepatitis B virus
HCC: Hepatocellular carcinoma
HR: Hazard ratio
LASSO: Least absolute shrinkage and selection operator
LSM: Liver stiffness measurement
OS: Overall survival
ROC: Receiver operating characteristic
TE: Transient elastography
tdAUC: Time-dependent area under the curve
VIF: Variance inflation factor
