# Supplementary material for: LEAST as a novel prediction model of hepatocellular carcinoma development in patients with chronic hepatitis B: a multi-center study
Source: BMC Med. 2025 Nov 3;23:603. doi: 10.1186/s12916-025-04430-2 (PMC12581542; doi:10.1186/s12916-025-04430-2)
Supplement: Supplementary file 1 — Additional file 1: Tables S1–S3. Table S1 Baseline characteristics of the external validation cohort. Table S2 Goodness of fit and collinearity diagnosis of model variables. Table S3 The differences in discriminative ability between all the predictive models and the LEAST model. [file 12916_2025_4430_MOESM1_ESM.docx]

**Additional file 1**

Table S1-Baseline characteristics of the external validation cohort.

Table S2 -Goodness of fit and collinearity diagnosis of model variables.

Table S3-The differences in discriminative ability between all the predictive models and the LEAST model.

**Table S1** Baseline characteristics of the external validation cohort

|  | External validation cohort 1 (n=1084) | External validation cohort 2 (n=623) | *P* |
| --- | --- | --- | --- |
| HCC (%) | 119 (10.98%) | 46 (7.38%) | 0.020 |
| Male (%) | 781 (72.05%) | 433 (69.50%) | 0.288 |
| Age (years) | 46.00 (35.00,55.00) | 47.00 (36.00,56.00) | 0.128 |
| PLT(10^9/L) | 154.00 (112.75,194.00) | 145.00 (134.00,192.00) | 0.267 |
| ALB (g/L) | 41.00 (36.00,44.00) | 44.00 (40.70,45.80) | ＜0.001 |
| LSM (kPa) | 12.40 (7.90,17.50) | 7.30 (5.80,11.80) | ＜0.001 |
| Alcohol use (%) | 81 (7.47%) | 63 (10.11%) | 0.072 |
| HBV DNA  (log_10_ IU/mL) | 4.21 (2.70,6.75) | 2.70 (1.30,4.22) | ＜0.001 |
| WBC (10^9/L) | 5.00 (4.13,6.09) | 4.97 (4.52,6.10) | 0.139 |
| HGB (g/L) | 145.00 (131.00,155.00) | 141.00 (135.00,154.50) | 0.534 |
| ALT (U/L) | 63.00 (32.75,225.00) | 28.90 (18.95,48.00) | ＜0.001 |
| AST (U/L) | 53.00 (31.00,138.00) | 25.90 (21.00,37.00) | ＜0.001 |
| TB (µmol/L) | 18.10 (13.00,28.33) | 13.60 (10.00,20.50) | ＜0.001 |
| ALP (U/L) | 95.00 (75.00,128.25) | 73.80 (60.75,90.35) | ＜0.001 |
| GGT (U/L) | 56.00 (30.00,119.00) | 26.00 (17.30,40.00) | ＜0.001 |
| TC (mmol/L) | 4.34 (3.72,4.95) | 4.09 (3.84,4.62) | ＜0.001 |
| TG (mmol/L) | 1.08 (0.82,1.46) | 0.94 (0.80,1.14) | ＜0.001 |
| GLU (mmol/L) | 5.15 (4.73,5.73) | 4.88 (4.64,5.15) | ＜0.001 |
| CREA (µmol/L) | 68.00 (60.00,76.00) | 63.00 (57.00,72.00) | ＜0.001 |
| AFP (ng/ml) | 6.20 (3.50,15.76) | 3.15 (1.90,4.21) | ＜0.001 |
| UAP (dB/m) | 228.00 (213.00,248.00) | 228.00 (210.00,256.00) | 0.717 |
| FIB-4 | 2.09 (1.18,4.55) | 1.51 (0.96,2.22) | ＜0.001 |
| APRI | 1.05 (0.47,2.73) | 0.42 (0.29,0.70) | ＜0.001 |
| C-index  95%CI | 0.807  0.772-0.842 | 0.905  0.862-0.948 |  |

HCC, hepatocellular carcinoma; WBC, white blood cell; HGB, hemoglobin; PLT, platelet; ALB, albumin; ALT, alanine transaminase; AST, aspartate aminotransferase; TB, total bilirubin; ALP, alkaline phosphatase; GGT, gamma-glutamyl transferase; TC, total cholesterol; TG, triglyceride; GLU, glucose; CREA, creatinine; AFP, alpha-fetoprotein; LSM, liver stiffness measurement; UAP, ultrasound attenuation parameter; CI, Confidence interval.

**Table S2** Goodness of fit and collinearity diagnosis of model variables

| Variable | VIF | Chisq | *P* |
| --- | --- | --- | --- |
| Sex | 1.039 | 1.129 | 0.29 |
| Age | 1.096 | 0.008 | 0.93 |
| PLT | 1.131 | 0.412 | 0.52 |
| ALB | 1.155 | 0.667 | 0.80 |
| LSM | 1.028 | 0.370 | 0.54 |
| GLOBAL | / | 2.858 | 0.72 |

ALB, albumin; PLT, platelet; LSM, liver stiffness measurement; Chisq: Chi-Square Test; VIF, Variance inflation factor.

**Table S3** The differences in discriminative ability between all the predictive models and the LEAST model

| *P* value | 3-year | 5-year | 8-year |
| --- | --- | --- | --- |
| Model B | 0.958 | 0.372 | 0.882 |
| Model C | 0.289 | 0.025 | 0.394 |
| Model D | 0.110 | <0.001 | 0.040 |
| Model E | 0.198 | 0.038 | 0.182 |
| Model F | 0.825 | 0.115 | 0.209 |

Model B: REAL-B, Model C: mREACH-B, Model D: AGED, Model E: mPAGE-B, Model F: LSM-HCC.
