# Supplementary material for: LEAST as a novel prediction model of hepatocellular carcinoma development in patients with chronic hepatitis B: a multi-center study
Source: BMC Med. 2025 Nov 3;23:603. doi: 10.1186/s12916-025-04430-2 (PMC12581542; doi:10.1186/s12916-025-04430-2)
Supplement: Supplementary file 2 — Additional file 2: Figures S1–S4. Fig. S1 (A) Variables selected by the LASSO regression. (B) Forest plots for multivariate analyses of risk factors concerning HCC development. LASSO, least absolute shrinkage and selection operator; HCC, hepatocellular carcinoma. Fig. S2 Applying the thresholds established by X-tile on the nomogram, the derivation cohort was divided into three risk categories. ALB, albumin; PLT, platelet; HR, hazard ratio; CI, confidence interval; LSM, liver stiffness measurement. Fig. S3 Kaplan–Meier curves for the external validation cohort 1, after dichotomizing the cohort using the risk-stratification cut-off value rounded to the nearest integer (low risk < 160 points, intermediate risk 160–193 points, high risk > 193 points). HCC, hepatocellular carcinoma. Fig. S4 Kaplan–Meier curves for the external validation cohort 2, after dichotomizing the cohort using the risk-stratification cut-off value rounded to the nearest integer (low risk < 160 points, intermediate risk 160–193 points, high risk > 193 points). HCC, hepatocellular carcinoma. Graphical Abstract—This graphical abstract illustrates the overall workflow for developing and validating a hepatocellular carcinoma prediction model. The process begins with a baseline study of a chronic hepatitis B cohort, followed by the construction of a statistical model, and concludes with successful validation across multiple independent external cohorts. [file 12916_2025_4430_MOESM2_ESM.docx]

**Additional file 2**

**Fig. S1-** (A) Variables selected by the LASSO regression, (B) Forest plots for multivariate analyses of risk factors concerning HCC development. LASSO, least absolute shrinkage and selection operator. HCC, hepatocellular carcinoma.

**Fig. S2-** Applying the thresholds established by X-tile on the nomogram, the derivation cohort was divided into three risk categories. ALB, albumin; PLT, platelet; HR, hazard ratio; CI, confidence interval; LSM, liver stiffness measurement.

**Fig. S3-** Kaplan–Meier curves for the external validation cohort 1, after dichotomizing the cohort using the risk-stratification cut-off value rounded to the nearest integer (low risk<160 points, intermediate risk 160-193 points, high-risk>193 points). HCC, hepatocellular carcinoma.

**Fig. S4**- Kaplan–Meier curves for the external validation cohort 2, after dichotomizing the cohort using the risk-stratification cut-off value rounded to the nearest integer (low risk<160 points, intermediate risk 160-193 points, high-risk>193 points). HCC, hepatocellular carcinoma.

**Graphical Abstract-**This graphical abstract illustrates the overall workflow for developing and validating a hepatocellular carcinoma prediction model. The process begins with a baseline study of a chronic hepatitis B cohort, followed by the construction of a statistical model, and concludes with successful validation across multiple independent external cohorts.


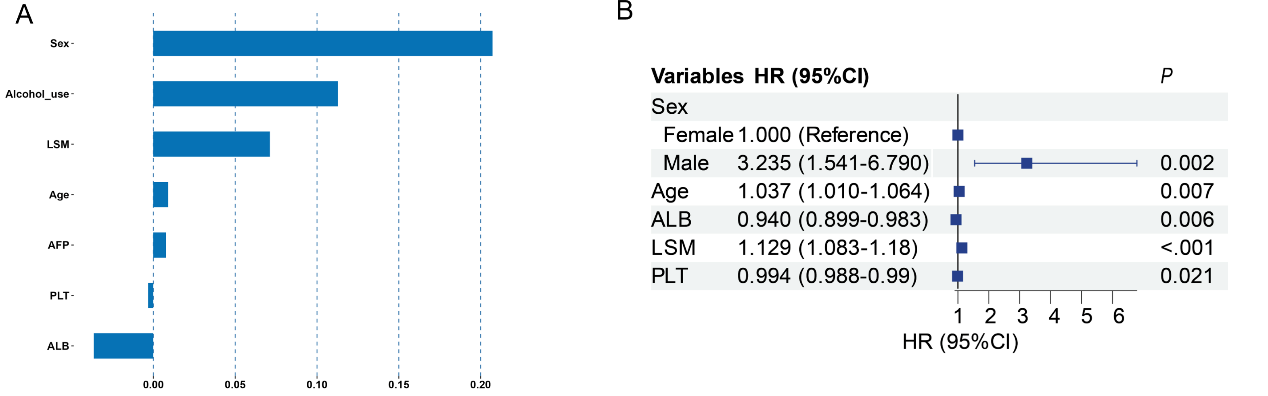


**Fig. S1** (A) Variables selected by the LASSO regression, (B) Forest plots for multivariate analyses of risk factors concerning HCC development. LASSO, least absolute shrinkage and selection operator. HCC, hepatocellular carcinoma.


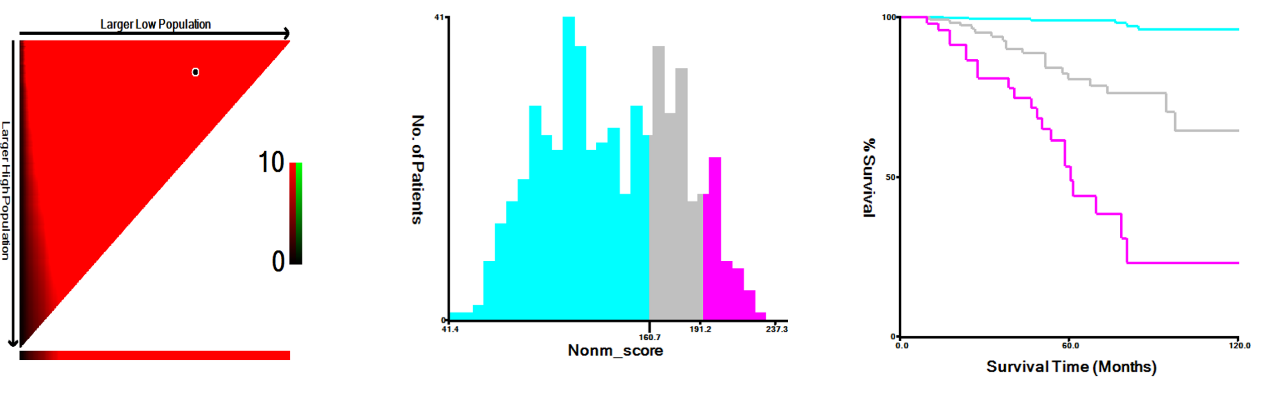


**Fig. S2** Applying the thresholds established by X-tile on the nomogram, the derivation cohort was divided into three risk categories. ALB, albumin; PLT, platelet; HR, hazard ratio; CI, confidence interval; LSM, liver stiffness measurement.

**
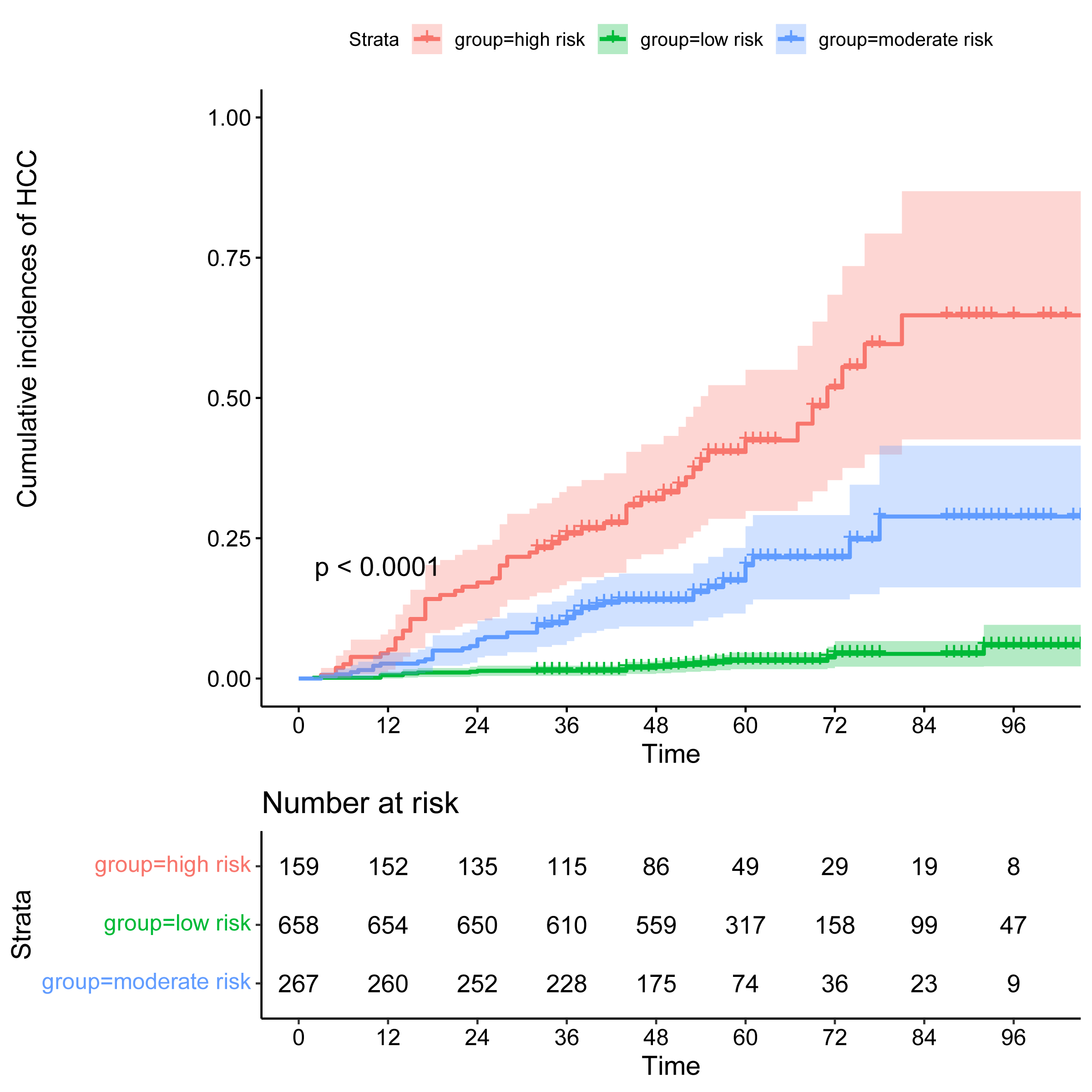
**

**Fig. S3** Kaplan–Meier curves for the external validation cohort 1, after dichotomizing the cohort using the risk-stratification cut-off value rounded to the nearest integer (low risk<160 points, intermediate risk 160-193 points, high-risk>193 points). HCC, hepatocellular carcinoma.


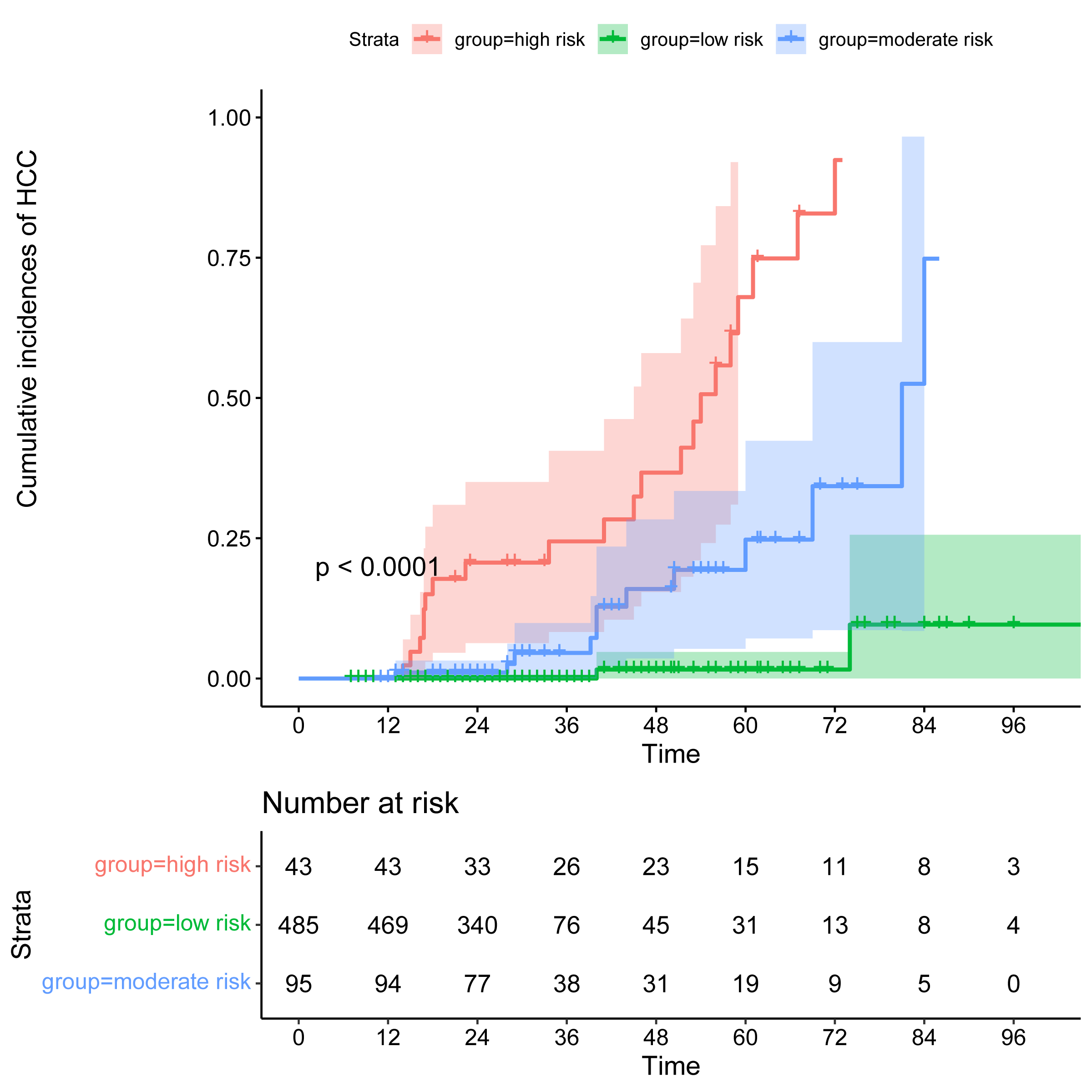


**Fig. S4** Kaplan–Meier curves for the external validation cohort 2, after dichotomizing the cohort using the risk-stratification cut-off value rounded to the nearest integer (low risk<160 points, intermediate risk 160-193 points, high-risk>193 points). HCC, hepatocellular carcinoma.


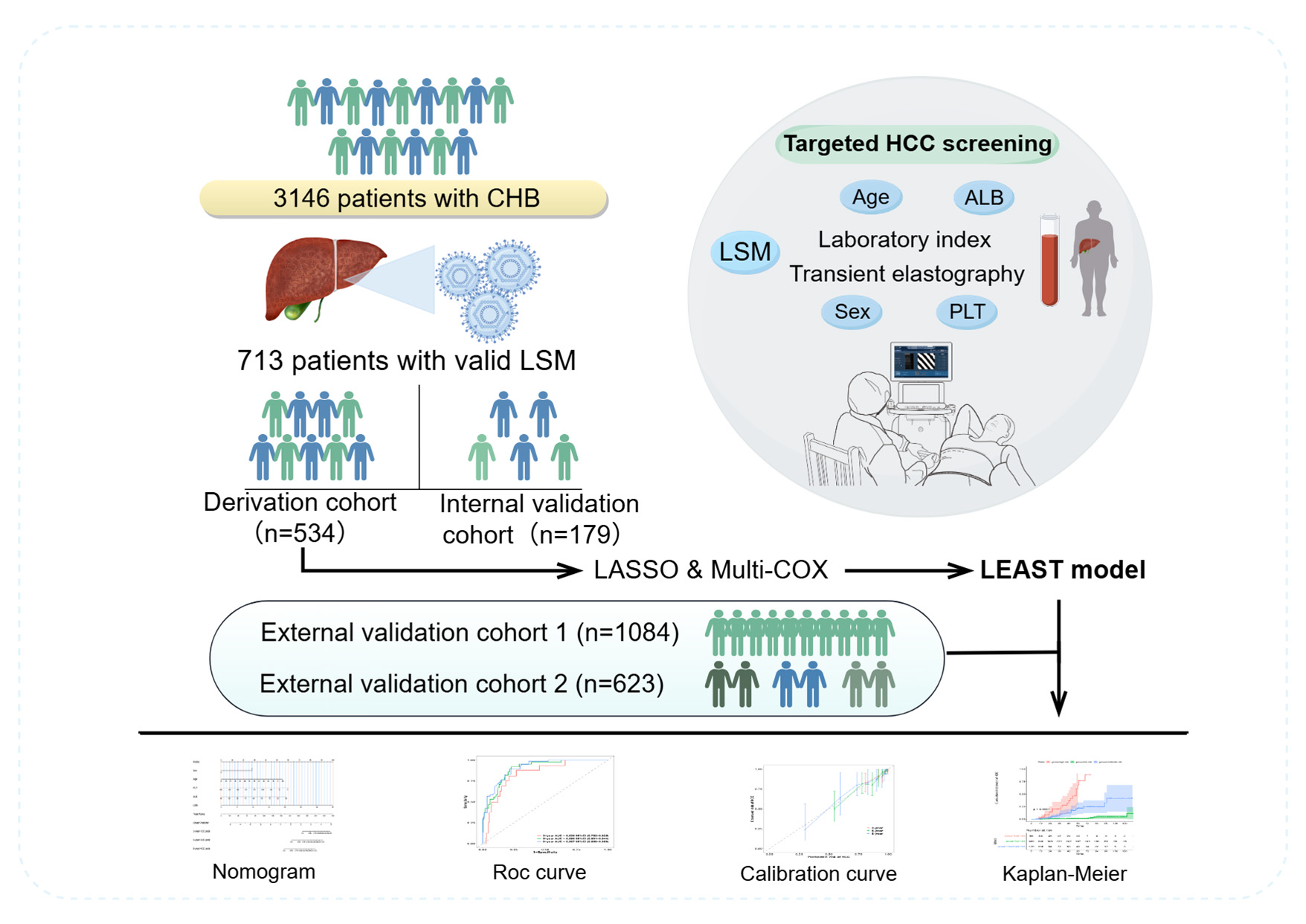


**Graphical Abstract**

This graphical abstract illustrates the overall workflow for developing and validating a hepatocellular carcinoma prediction model. The process begins with a baseline study of a chronic hepatitis B cohort, followed by the construction of a statistical model, and concludes with successful validation across multiple independent external cohorts.
